# Elevational variation in body-temperature response to immune challenge in a lizard

**DOI:** 10.7717/peerj.1972

**Published:** 2016-04-25

**Authors:** Francisco Javier Zamora-Camacho, Senda Reguera, Gregorio Moreno-Rueda

**Affiliations:** 1 Department of Biological Sciences, Dartmouth College, Hanover, NH, United States; 2 Departamento de Zoología, Universidad de Granada, Granada, Spain

**Keywords:** Cost/benefit balance, Ectotherm, Elevation, Immune system, *Psammodromus algirus*, Thermoregulation

## Abstract

Immunocompetence benefits animal fitness by combating pathogens, but also entails some costs. One of its main components is fever, which in ectotherms involves two main types of costs: energy expenditure and predation risk. Whenever those costs of fever outweigh its benefits, ectotherms are expected not to develop fever, or even to show hypothermia, reducing costs of thermoregulation and diverting the energy saved to other components of the immune system. Environmental thermal quality, and therefore the thermoregulation cost/benefit balance, varies geographically. Hence, we hypothesize that, in alpine habitats, immune-challenged ectotherms should show no thermal response, given that (1) hypothermia would be very costly, as the temporal window for reproduction is extremely small, and (2) fever would have a prohibitive cost, as heat acquisition is limited in such habitat. However, in temperate habitats, immune-challenged ectotherms might show a febrile response, due to lower cost/benefit balance as a consequence of a more suitable thermal environment. We tested this hypothesis in *Psammodromus algirus* lizards from Sierra Nevada (SE Spain), by testing body temperature preferred by alpine and non-alpine lizards, before and after activating their immune system with a typical innocuous pyrogen. Surprisingly, non-alpine lizards responded to immune challenge by decreasing preferential body-temperature, presumably allowing them to save energy and reduce exposure to predators. On the contrary, as predicted, immune-challenged alpine lizards maintained their body-temperature preferences. These results match with increased costs of no thermoregulation with elevation, due to the reduced window of time for reproduction in alpine environment.

## Introduction

Immunocompetence influences animal fitness by combating pathogens (Schmid-Hempel, 2011). Nevertheless, deploying an efficient immune response is known to entail a number of costs (Lochmiller & Deerenberg, 2000; Demas, 2004; Schmid-Hempel, 2011), which force animals to trade off immune defence with other costly traits (Sheldon & Verhulst, 1996; Norris & Evans, 2000; Zuk & Stoehr, 2002). These costs imply that other life-history traits may be impaired by the activation of the immune system, such as breeding success (Råvberg et al., 2000), learning capacity (Grindstaff, Hunsaker & Cox, 2012), growth (Uller, Isaksson & Olsson, 2006), sexual attractiveness (López, Gabirot & Martín, 2009), and even survival (Moret & Schmid-Hempel, 2000; Hanssen et al., 2004; Eraud, Jacquet & Faivre, 2009).

In addition to humoral and cellular responses, one of the main components of immune response in most animals -endotherms as well as ectotherms (reviewed in Bicego, Barros & Branco, 2007)- is fever, a rise of body temperature with beneficial effects on other components of the immune system, such as the expression of genes involved in pathogen combating (Boltaña et al., 2013), and/or direct negative effects on pathogens (Kluger et al., 1998). The efficacy of fever against pathogens has long been documented in both endotherms (Hasday, Fairchild & Shanholtz, 2000) and ectotherms (Kluger, Ringler & Anver, 1975; Elliot et al., 2005). Hence, survival following infections is well known to increase with body temperature in both endotherms (Kluger et al., 1998) and ectotherms (Kluger, 1979). Nevertheless, the costs of producing fever and the optimal response considering these costs have received little attention (Roe & Kinney, 1965; Romanovsky & Székely, 1998), particularly in ectotherms. Endotherms generate heat endogenously, and thus fever implies increased energy expenditure (Marais, Maloney & Gray, 2011). In turn, ectotherms regulate body temperature mainly behaviourally, by exposing themselves to environmental heat sources (Belliure & Carrascal, 1998). This leads to two widespread and well-documented types of costs: (1) energy expenditure, as ectotherm metabolic rates escalate with body temperature (Gillooly et al., 2001), and (2) predation risk, as ectotherms’ mechanisms to gain heat, such as basking, usually imply an elevated exposure to predators (Herczeg et al., 2008). Therefore, under certain circumstances, the costs of raising body temperature might outweigh the immunological benefits of fever. In such cases, no thermal response to pathogens may occur. Animals may even show hypothermia in order to save energy (Deen & Hutchison, 2001).

Maintaining optimal temperature is well known to be energetically costly (Bennett & Ruben, 1979; Alford & Lutterschmidt, 2012; Brewster, Sikes & Gifford, 2013). Moreover, deploying an immune response (i.e., mounting any kind of immunological response to an immune challenge) is also energetically costly, even without fever. For example, in *Rhinella marinus* toads maintained at the same temperature, those injected with an antigen showed increased metabolic costs as a consequence of immune response (Sherman & Stephens, 1998). Indeed, although fever is a common response to pathogens, some endotherm as well as ectotherm vertebrates respond to antigens with hypothermia (Owen-Ashley et al., 2006; Merchant et al., 2008; King & Swanson, 2013). Since survival following infections usually decreases at lower temperatures (Kluger et al., 1998, and references therein), these findings also suggest a trade-off between rising, or even maintaining, body temperature, and deploying a leukocite-mediated immune response. In this scenario, on the one hand, body temperature could decrease in immune-challenged ectotherms (hypothermia). On the other hand, an increase in body temperature (fever) might also be expected in response to an immune challenge, as fever is known as an efficient defence against pathogens in ectotherms (Kluger, Ringler & Anver, 1975; Elliot et al., 2005). Therefore, fever, the absence of thermal response, or hypothermia in response to an immune challenge will depend on the cost/benefit balance of thermoregulation. Animals in a scenario of high thermoregulation cost/benefit balance might respond to an immune challenge with hypothermia, while animals in which costs of hyperthermia are low, or benefits are high, might respond to immune challenge with fever. Supporting this hypothesis, febrile or hypothermic response of iguana (*Iguana iguana*) juveniles to an activation of the immune system depends on the energetic state of each individual, only those individuals in the best energetic condition developing fever (Deen & Hutchison, 2001).

Given that ectotherms depend mainly on environmental temperature to thermoregulate, and environment thermal quality varies geographically (Sunday, Bates & Dulvy, 2010), the cost/benefit balance of producing fever should also vary geographically, especially at different elevations. Several physical and climatic variables follow general elevational patterns: ultraviolet radiation is greater (Reguera et al., 2014), while environment temperature is lower (Zamora-Camacho, Reguera & Moreno-Rueda, 2015) in alpine than in non-alpine areas. Environment temperature is expected to have a greater impact on ectotherm thermal response to immune challenge. On the one hand, the fact that thermal quality of the habitat for ectotherms typically decreases with elevation (Hertz & Huey, 1981) makes ectotherms spend more time basking in alpine zones (Díaz, 1997); therefore, the cost of thermoregulation increases with elevation. Accordingly, we predict that ectotherms in alpine zones should not develop fever in response to an immune challenge. On the other hand, although ectotherms may be expected to thermoconform (i.e., to lack thermoregulation behavior) when the costs of thermoregulation are high (Huey & Slatkin, 1976), thermoconformity in extremely cold habitats could lead to body temperatures too low for physiological processes such as reproduction (Stevenson, 1985; Herczeg et al., 2003). In fact, the window of time for reproduction declines with rising elevation, both in a daily and a yearly basis (Zamora-Camacho et al., 2013; Zamora-Camacho, Reguera & Moreno-Rueda, 2015), which may severely compromise ectotherm opportunities to accomplish their reproductive cycle, thus favoring thermoregulation in thermally-challenging habitats (Herczeg et al., 2003; Zamora-Camacho, Reguera & Moreno-Rueda, 2015). Consequently, the costs of no thermoregulation may outweigh those of thermoregulation in cold climates, in terms of loss of reproduction opportunities. Therefore, we hypothesize that, everything being equal, at contrasting elevations, alpine immune-challenged ectotherms should prioritize heat acquisition, and thus their body temperature should be maintained in comparison with non-alpine immune-challenged ectotherms. In other words, given that body temperature in alpine ectotherms is constrained by low thermal quality (precluding fever) as well as by short time for reproduction (precluding hypothermia), we predict that immune-challenged alpine ectotherms should not show thermal response to an immune challenge. By contrast, we predict that non-alpine ectotherms challenged with an antigen will show a febrile response, thanks to the higher thermal availability, which will reduce the cost/benefit balance.

In this work, we test this hypothesis by comparing the effect of an immune challenge on the temperature selected by a lizard, the large Psammodromus (*Psammodromus algirus*; C. Linnaeus 1758), from alpine (2,200–2,500 m asl (metres above sea level)) and non-alpine (300–1,700 m asl) zones on a mountain. In this study system, alpine lizards face a colder environment than do non-alpine lizards, but despite the differences in thermal environment, they keep field body temperatures similar to those of non-alpine lizards (Zamora-Camacho et al., 2013). Alpine lizards in this study area are, indeed, active at lower temperatures than non-alpine lizards, which allows them to complete their life cycle in the small window of time available in alpine areas (Zamora-Camacho et al., 2013). Accordingly, we predict that, in common-garden laboratory conditions of similar thermal availability, alpine-lizard thermal response (hypothermia or fever) to a typical pyrogen will be less intense or non-existent, as a consequence of their elevated costs of fever (low-thermal-quality environment) and of hypothermia (small window of time for reproduction), as a result of lower thermal availability in their origin populations (at both individual and evolutionary time scales).

## Materials and Methods

*Psammodromus algirus* is a medium-sized (53–95 mm of snout-vent length [SVL] in our study zone) Lacertid lizard that inhabits shrubby areas in the Western Mediterranean, from the sea level to more than 2,600 m asl (Salvador, 2011). Lizards were captured in Sierra Nevada ($36\textdegree 5{6}^{^{\prime}}\mathrm{N}$ $3\textdegree 2{3}^{^{\prime}}\mathrm{O}$; SE Spain), during their reproductive season (April–July) in 2012. As a part of a long-term project, the study area was divided into six sampling plots distributed approximately every 500 m of elevation, four of them placed in non-alpine zones (300, 700, 1,200, and 1,700 m asl), the remaining two in alpine zones (2,200 and 2,500 m asl, respectively). Climatic and biotic conditions support this grouping. In alpine zones, winter precipitation takes the form of snow, which usually covers the soil during six months a year on average (November–May); summers are moderately warm, and average daily temperatures during lizard activity period are below 19 °C (Mean ± SE; 2,200 m asl: 16.54 ± 0.64 °C; 2,500 m asl: 17.22 ± 0.78 °C). Alpine vegetation is well adapted to those harsh climatic conditions: forests are open and composed of conifers, the timberline appears at roughly 2,300 m asl, and shrubs, where trees lack, are dense and small. By contrast, in non-alpine zones, precipitation typically takes the form of rain, winter snowfalls being occasional or absent; summers are hot, and average daily temperatures during lizard activity period are above 19 °C (Mean ± SE; 300 m asl: 25.02 ± 0.59 °C; 700 m asl: 23.01 ± 0.58 °C; 1,200 m asl: 20.87 ± 0.60 °C; 1,700 m asl: 19.08 ± 0.61 °C). Predominant vegetation in non-alpine zones consists of Mediterranean sclerophyllous forests and associated shrubbery (more details in Appendix A in Zamora-Camacho et al., 2013).

We took lizards (15 from alpine elevations and 18 from non-alpine elevations) to the laboratory, and measured body mass with a balance model CDS-100 (accuracy 0.01 g). The first day in the lab (day 1), we placed lizards into individual terrariums (100 × 20 × 40 cm), built using 0.5 mm-thick methacrylate, with a 150W red-light bulb on one side, 15 cm above the pine-bark substrate. Bulbs were lit during daytime, generating a 20–55 °C linearly-arrayed temperature gradient, which covers the temperatures usually preferred by this lizard (Díaz 1997). A window provided natural light-darkness cycles, so that lizards could adjust their circadian rhythms. Being left undisturbed during the entire day, all the lizards spent the same time in the same environment before trials, independently of their origin, thereby avoiding differences that could be ascribed to thermal conditions (Deen & Hutchison, 2001).

On day 2, lizards were allowed to thermoregulate during the entire day, and we registered their preferred temperatures $({T}_{\mathrm{pref}})$ once per hour, from 10:00 to 14:00 h (local time). Body temperature was measured with a small catheter connected to a thermometer (Hybok 18, accuracy 0.1 °C) and inserted 8 mm inside the cloaca. Each time we handled a lizard, it was returned to the centre of the terrarium with the body aligned perpendicularly to the thermal gradient. We did so in order to avoid an effect of lizard position on body-temperature preferences (Llewellyn et al., 2013).

On day 3, lizards were randomly assigned to experimental or control groups. At 9:45 h, lizards in the experimental group were inoculated subcutaneously in the hind sole pad with 0.1 mg of lipopolysaccharide (LPS) of bacterial wall of *Escherichia coli* (serotype 055:B5, L-2880, Sigma Aldrich), diluted in 0.01 ml of phosphate buffer. Meanwhile, control lizards were inoculated with 0.01 ml of isotonic phosphate buffered saline (PBS), which has no physiological effects. LPS is an innocuous antigen that stimulates the immune system, without other effects on the organism, provoking an immune response that peaks four hours after inoculation (Parmentier, De Vries Reilingh & Nieuwland, 1998). LPS is probably the most frequent pyrogen used in studies on fever (Kluger, 1991). To evaluate the response to the antigen, we measured the thickness of the inoculated sole pad using a pressure-sensitive micrometer (Mitutoyo; accuracy 0.01 mm) immediately before and four hours after injecting the substances, in lizards from both LPS and PBS groups. The degree of inflammation of the region injected is directly related to leukocyte concentration (Parmentier, De Vries Reilingh & Nieuwland, 1998). We took three sole-pad thickness measurements each time, and calculated the average value, the difference being the immune-response magnitude. Immediately after the inoculations, lizards were individually reallocated in the temperature-gradient terrariums (as described above), and we recorded their body temperature once per hour from 10:00 to 14:00 h (local time). Thus, ${T}_{\mathrm{pref}}$ was registered while lizards were developing the immune response. Then, we calculated the average values of the five ${T}_{\mathrm{pref}}$ measurements on day 2 (before inoculation) and day 3 (after inoculation), as well as the difference between the two measurements.

Since immune challenge reduces female lizard reproductive output (Uller, Isaksson & Olsson, 2006), we used only adult males for this experiment, in order to avoid interfering with ovogenesis and gestation. We identified males because they have proportionally larger heads, orange mouth commissures, and more prominent femoral pores. Also, since testosterone affects immune response in this species (Belliure, Smith & Sorci, 2004), lizards were captured only during their reproductive season at each sampling plot (April–July in the four lowest sampling plots, May–July in the two highest sampling plots), in order to avoid seasonal shifts in plasma testosterone concentration. Plasma testosterone concentration is usually higher during reproductive season than afterwards (Tokarz et al., 1998; Wack et al., 2008; Gowan, McBrayer & Rostal, 2010). During their stay in captivity, lizards were provided *ad libitum* with mealworms (*Tenebrio molitor* larvae) and water (in form of a nutritious aqueous gel). Once the experiment ended, lizards were returned to their place of capture. The experiments comply with the current laws of Spain, and were performed in accordance with Junta de Andalucía research permits (references GMN/GyB/JMIF and ENSN/JSG/JEGT/MCF).

As the data fulfilled the criteria of residual normality and homoscedasticity (Quinn & Keough, 2002), we used parametric statistics. We conducted Linear Mixed Models of Restricted Maximum Likelihood (REML-LMM; Zuur et al., 2009), by using the package “nlme” (Pinheiro et al., 2012) in software R (R Development Core Team, 2012). Population of origin was introduced as a random factor, therefore correcting for any possible pseudoreplication, and we tested for the effect of treatment, elevation (non-alpine vs. alpine origin of the lizards), and its interaction on dependent variables. Firstly, we used the aforesaid model to check differences in body mass or initial ${T}_{\mathrm{pref}}$ according to treatment and elevation. In addition, we tested for the effect of treatment, elevation, and their interaction on immune-response magnitude (change in sole-pad thickness before and after trials). We then performed two separate models, for alpine and non-alpine lizards, in order to test the effect of treatment on difference in ${T}_{\mathrm{pref}}$ (final ${T}_{\mathrm{pref}}$ minus initial ${T}_{\mathrm{pref}}$). Then, we tested the effect of treatment, elevation, and its interaction on final ${T}_{\mathrm{pref}}$, controlling for initial ${T}_{\mathrm{pref}}$ (included as a covariate). We predicted an effect of treatment on the final ${T}_{\mathrm{pref}}$, as well as a significant treatment*elevation interaction. Finally, we conducted correlations between sole-pad swelling and post-inoculation ${T}_{\mathrm{pref}}$ in alpine and non-alpine LPS-inoculated lizards. In all models, we discarded including body mass as a covariate in order to avoid collinearity, because body mass was correlated with elevation (*r* = 0.567; *P* < 0.001). We visually checked that residuals of the models were normally distributed, and tested that they accomplished the criterions of homoscedasticity with the Levene’s test (Zuur, Ieno & Elphick, 2010).

**Figure 1:**
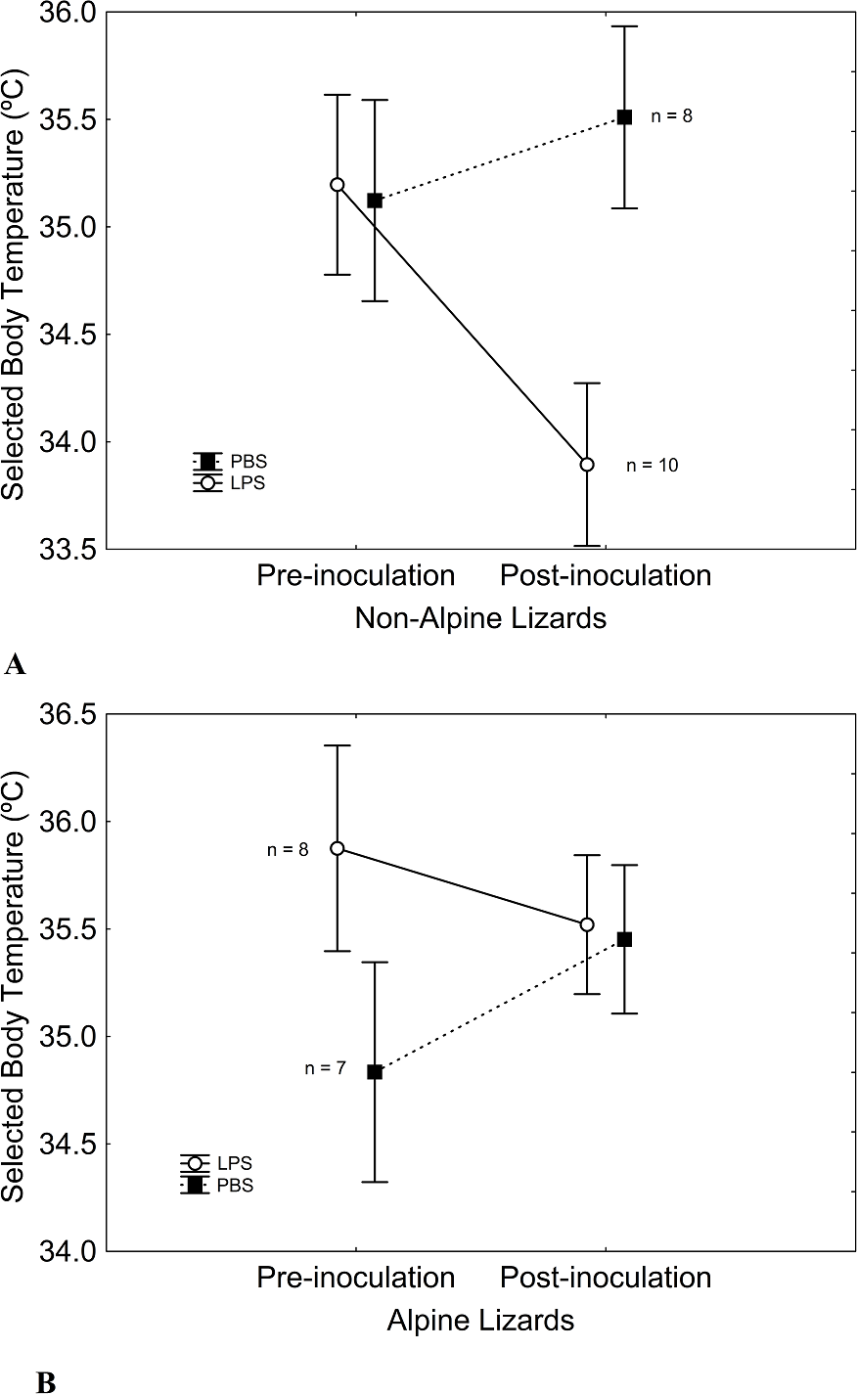
Lizard ${T}_{\mathrm{pref}}$ before and after inoculations for each elevation belt regarding treatment. All lizards showed similar preferred body-temperature $({T}_{\mathrm{pref}})$ before inoculations. After inoculations, a trade-off between ${T}_{\mathrm{pref}}$ and immune system appeared in non-alpine lizards (Fig. 1A), since LPS-inoculated lizards showed lower ${T}_{\mathrm{pref}}$ than did PBS-inoculated lizards. Nevertheless, this trade-off did not affect alpine lizards (Fig. 1B), which selected similar temperatures after inoculations regardless of the substance inoculated. Vertical bars represent standard errors.

**Table 1:** Average values ± standard errors of body mass, preferential body temperature $({T}_{\mathrm{pref}})$ before and after the inoculations, and inflammation response (sole-pad swelling in mm), for both groups (LPS and PBS), as well as for alpine (2,200–2,500 m asl) and non-alpine (300–1,700 m asl) lizards. Sample size is shown in parentheses. Differences between treatments and elevations were tested with a REML-LMM with population as random factor. For post-inoculation ${T}_{\mathrm{pref}}$, we controlled for pre-inoculation ${T}_{\mathrm{pref}}$, included as covariate (*χ*^2^ = 2.75, *P* = 0.097).

Variable	LPS group (*n* = 18)	PBS group (*n* = 15)	Alpine (*n* = 15)	Non-alpine (*n* = 18)	Effect of treatment	Effect of elevation	Treatment^*^ Elevation
**Body mass** (g)	7.41 ± 0.64	6.88 ± 0.70	8.95 ± 0.55	5.68 ± 0.50	*χ*^2^ = 1.46	${\chi }^{\mathbf{2}}=\mathbf{6.80}$^**^	*χ*^2^ = 0.84
Pre-inoculation ${T}_{\mathrm{pref}}$ (°C)	35.50 ± 0.31	34.99 ± 0.34	35.39 ± 0.35	35.16 ± 0.32	*χ*^2^ = 0.01	*χ*^2^ = 1.13	*χ*^2^ = 1.06
Post-inoculation ${T}_{\mathrm{pref}}$ (°C)	34.62 ± 0.32	35.48 ± 0.26	35.49 ± 0.31	34.61 ± 0.29	${\chi }^{\mathbf{2}}=\mathbf{10.77}$^**^	${\chi }^{\mathbf{2}}=\mathbf{8.30}$^**^	${\chi }^{\mathbf{2}}=\mathbf{3.74}$^§^
Footpad swelling (mm)	0.08 ± 0.02	−0.02 ± 0.02	0.02 ± 0.02	0.05 ± 0.03	${\chi }^{\mathbf{2}}=\mathbf{8.06}$^**^	*χ*^2^ = 0.30	*χ*^2^ = 0.10

**Notes.**

In bold significant or almost significant results.

§ *P* = 0.053.

* *P* < 0.05.

** *P* < 0.01.

**Figure 2:**
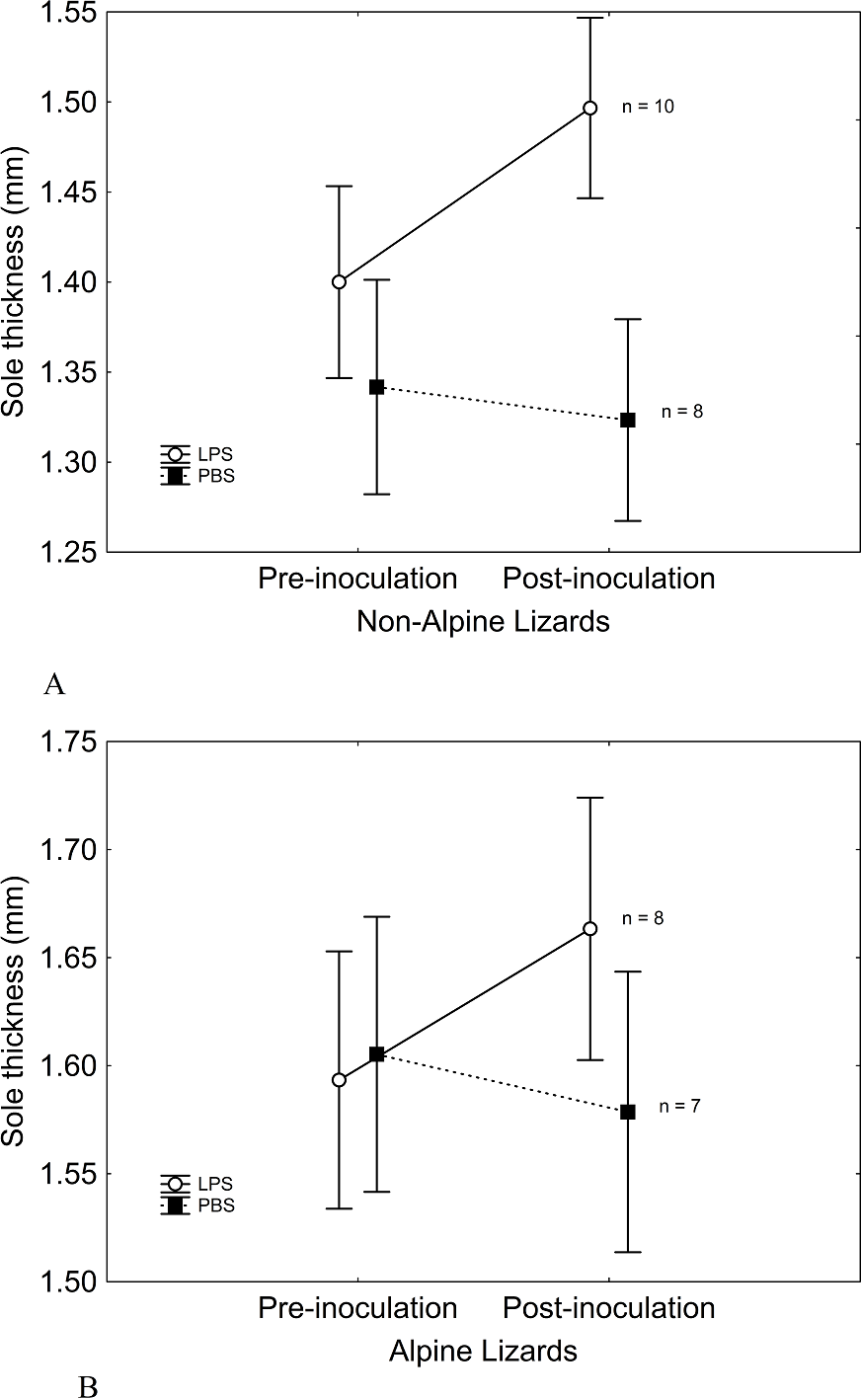
Sole-pad thickness was similar in both LPS and PBS-inoculated lizards. Four hours after the inoculation, sole-pad thickness of PBS-inoculated lizards did not change significantly, while an inflammation occurred in LPS-inoculated lizards, pointing to an actual physiological effect of LPS. Patterns were similar in non-alpine lizards (Fig. 2A) and alpine lizards (Fig. 2B). Vertical bars represent standard errors.

**Figure 3:**
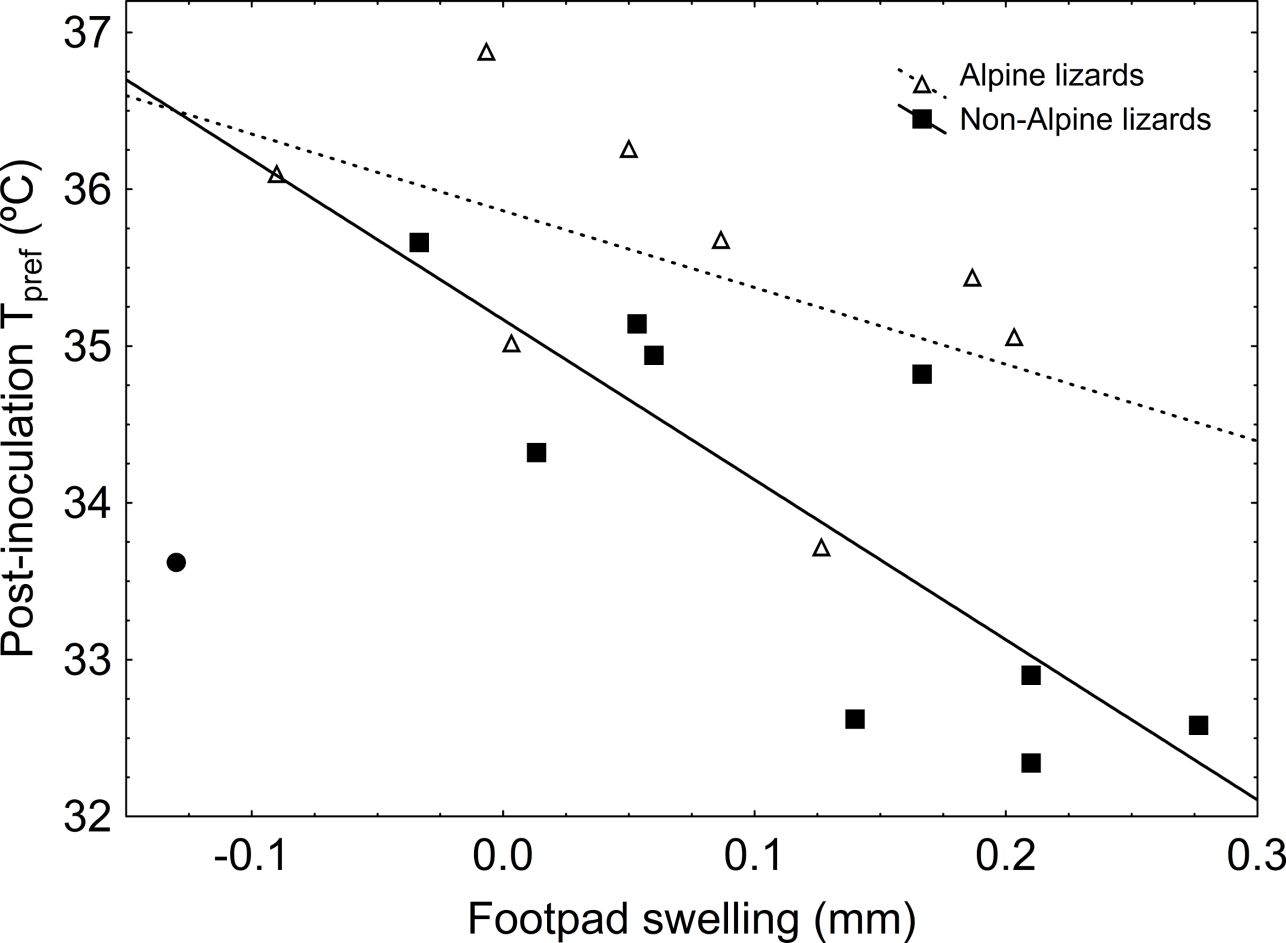
Correlations between sole-pad swelling and post-inoculation ${T}_{\mathrm{pref}}$ in alpine and non-alpine LPS-inoculated lizards. Lizards that showed the highest immune response selected the lowest ${T}_{\mathrm{pref}}$ after the inoculation, supporting that the change in ${T}_{\mathrm{pref}}$ was a consequence of immune response. As expected, correlation skimmed significance (and was indeed significant when an outlier -solid circle in the figure- was removed) only in non-alpine lizards, which evidenced a thermal response to inoculation of LPS, whilst it was non-significant in alpine lizards, which developed no thermal response to immune challenge. Non-alpine lizard line represents the correlation excluding the aforesaid outlier. Note that, although alpine and non-alpine lizards showed no difference in ${T}_{\mathrm{pref}}$ before the treatment, after the inoculation of LPS, non-alpine lizards showed lower ${T}_{\mathrm{pref}}$ than alpine lizards.

## Results

We found no significant differences between treatments in body mass or ${T}_{\mathrm{pref}}$ prior to inoculations (Fig. 1 and Table 1). Alpine lizards were larger than non-alpine lizards, but showed similar ${T}_{\mathrm{pref}}$ before the treatment (Fig. 1 and Table 1). The treatment was effective in stimulating an inflammatory immune response, as evidenced by a significant swelling of the sole pad in lizards inoculated with LPS, while lizards inoculated with PBS showed no change in the thickness of their sole pad (Table 1 and Fig. 2). Elevation and its interaction with treatment had no significant effect on sole pad swelling. Non-alpine LPS-inoculated lizards showed a ${T}_{\mathrm{pref}}$ significantly lower than their own ${T}_{\mathrm{pref}}$ before the inoculation, that is, difference in ${T}_{\mathrm{pref}}$ was significantly greater in LPS- than in PBS-inoculated non-alpine lizards (*F*_1,16_ = 5.73; *P* = 0.02). However, this effect was non-significant in alpine lizards (*F*_1,13_ = 2.18; *P* = 0.14). Also, treatment had a significant effect on post-inoculation ${T}_{\mathrm{pref}}$. Summarizing the main findings, although pre-inoculation ${T}_{\mathrm{pref}}$ did not differ between LPS- and PBS-inoculated lizards, or alpine and non-alpine lizards (above), non-alpine LPS-inoculated lizards showed a ${T}_{\mathrm{pref}}$ significantly lower than non-alpine PBS-inoculated lizards (Fig. 1A and Table 1). However, immune-challenged alpine lizards did not show lower ${T}_{\mathrm{pref}}$ than control (Table 1 and Fig. 1B). The interaction Treatment*Elevation skimmed significance (*χ*^2^ = 3.74, *P* = 0.053) when controlling for initial ${T}_{\mathrm{pref}}$, but was significant when initial ${T}_{\mathrm{pref}}$ was removed of the model (*χ*^2^ = 4.93, *P* = 0.026). The elevational difference in ${T}_{\mathrm{pref}}$ in LPS-inoculated lizards cannot be ascribed to differences in immune response, as the magnitude of immune response to LPS of alpine and non-alpine lizards was similar (Table 1). Moreover, considering only LPS-inoculated non-alpine lizards, the only ones that evidenced a thermal response to immune challenge, those showing greater swelling response to LPS also showed a marginally non-significant trend to lower post-inoculation ${T}_{\mathrm{pref}}$ (*r* = − 0.58, *P* = 0.079). Such trends became significant when a possible outlier with a very low inflammatory response was removed (*r* = − 0.81, *P* = 0.008, Fig. 3). As for alpine lizards, which evidenced no thermal response to immune challenge, the correlation between swelling and thermal response was non-significant, as expected (*r* = − 0.51, *P* = 0.26; Fig. 3).

## Discussion

In this study, we found that the thermal response of a lizard to an immune challenge varied at contrasting elevations. Immune-challenged alpine lizards showed ${T}_{\mathrm{pref}}$ similar to control lizards, presumably because, in the environment where they have evolved, their capacity to raise body temperature is constrained by the costs of thermoregulation (precluding fever), whilst the costs of non-thermoregulation (reduced reproduction opportunities) were so high that they could not afford to suppress thermoregulation (precluding hypothermia). Surprisingly, contrary to expected, non-alpine immune-challenged lizards showed a reduction in ${T}_{\mathrm{pref}}$, which evidences a plastic (depending on environment thermal availability) trade-off between thermoregulation and leukocyte-mediated immune response, regarding the benefits and the costs of hypothermia in an immune-challenge context.

These findings cannot be ascribed to differences in the magnitude of leukocyte-mediated immune response to LPS between alpine and non-alpine lizards, as such magnitude, ascertained as sole pad swelling, was similar in both and the change in ${T}_{\mathrm{pref}}$ was correlated with the magnitude of sole pad swelling. Moreover, the similar magnitude of sole pad swelling in alpine and non-alpine lizards suggests that the dose of LPS injected was appropriate to induce an immune response of similar strength in both elevations. Supporting this affirmation, in a different study we found that the inoculation of the same dose of LPS used in the current work had an impact on escape speed in male *P. algirus* in our study system, but escape speed was reduced in a similar degree in every elevation (Zamora-Camacho et al., 2015). As a whole, these findings suggest that the dosage used here has no mass-dependent effects on the immune system: it decreases escape speed and provokes swelling similarly across elevations, but the thermal response was elevation-dependent.

Also, our results cannot be attributed to acclimation to different thermal conditions, which may affect the response to pyrogens (Deen & Hutchison, 2001), given that lizards were acclimated to the same conditions in the laboratory. In fact, lizards showed the same ${T}_{\mathrm{pref}}$ during the control phase regardless of their elevational origin. In the field, body temperature does not differ with elevation, either (Zamora-Camacho et al., 2013; Zamora-Camacho, Reguera & Moreno-Rueda, 2015). Therefore, the findings in this study suggest that, as we predicted, lizards with a different background on thermal availability (both at individual and evolutionary timescales) use different thermal response to pathogens according to elevation when supplied with the same thermal availability.

Why did immune-challenged non-alpine lizards decrease their ${T}_{\mathrm{pref}}$ rather than showing fever, despite higher thermal availability? Leukocyte-mediated immune response is energetically costly (review in Schmid-Hempel, 2011), and high body temperature implies raised metabolism, with the concomitant expenditure of resources (Gillooly et al., 2001). In fact, inoculation of LPS in ectotherms increases metabolic expenditure (Sherman & Stephens, 1998). Therefore, we propose that LPS-inoculated lizards that selected lower body temperature diminished the energetic costs associated with metabolism, diverting the energy saved to the immune system. This assertion is supported by the negative correlation between the strength of the swelling immune response and body temperature. Furthermore, in ectotherms, a higher body temperature implies more prolonged basking periods, and therefore greater exposition to predators (Herczeg et al., 2006). Male *P. algirus* in this system show impaired escape speed when immune-challenged (Zamora-Camacho et al., 2015b), so their chances to evade predator attacks could be reduced in such a situation. In addition, at low elevation, fever might be constrained by the high risk of overheating (Zamora-Camacho, Reguera & Moreno-Rueda, 2015; also see Sköld-Chiriac et al., 2015). In fact, hypothermia is not an infrequent response to immune challenges (Deen & Hutchison, 2001; Do Amaral, Marvin & Hutchison, 2002; Owen-Ashley et al., 2006; Merchant et al., 2008; King & Swanson, 2013; and references therein), although its evolutionary importance has received little attention in comparison with fever. Consequently, in consideration of the costs and benefits involved in a thermal response to pathogens, fever may not always be the best option. However, we cannot discard the possibility that immune-challenged lizards might develop fever beyond 4 h from infection, since that is the duration of our experiment. In fact, small endotherms have been found to develop a biphasic response to immune challenge, where initial hypothermia was followed by fever (Derijk et al., 1994).

Hypothermia may also occur in immune-challenged non-alpine lizards because it helps them to combat parasites. Although the benefits of fever against pathogens are well established, and improved survival associated with fever in infected lizards has been demonstrated (Kluger, Ringler & Anver, 1975), some parasites, such as haemogregarines, undergo reduced multiplication rates at lower temperatures (Oppliger, Célérier & Clobert, 1996). Therefore, decreased body temperature could be an adaptive response against some pathogens. This explanation, nevertheless, seems improbable, given that haemogregarines prevalence is greater at high elevation than at low elevation in our population (Álvarez-Ruiz, 2015). Therefore, if parasites mediated the geographic variation in this study, we would expect just the reverse finding.

As a third possibility, immune-challenged lizards’ thermoregulation capability could be impaired due to sickness behaviour (Llewellyn et al., 2013). Sickness behaviour is usually elicited as a consequence of immune-system activation, and it implies diminished motility (Adelman & Martin, 2009), as physical exertion may reduce the ability to mount an immune response (Lopes, Springthorpe & Brently, 2014). Therefore, sick lizards could not be able to select the right position to thermoregulate. Nonetheless, in this case (no thermoregulation), we would expect higher variance in body temperature in LPS-inoculated lizards. We tested the differences in variance in post-inoculation ${T}_{\mathrm{pref}}$ between LPS and PBS lizards from the non-alpine zone, and found no significant differences (Levene test, *F*_1,16_ = 2.61, *P* = 0.13). Consequently, our findings cannot be ascribed to differences in sickness behaviour. Moreover, we visually checked, with a random periodicity, that lizards moved normally within terraria during the experiments, and detected no sign of impaired motility or any other symptom of sickness behaviour (personal observations).

In any case, lower body temperature in immune-challenged lizards would have a number of detrimental effects on fitness derived either from lower motility, such as reduced mating opportunities, territory loss or reduced food intake, or from physiological constraints, such as longer food-passage time (Van Damme, Bauwens & Verheyen, 1991; Chen, Xu & Ji, 2003; Llewellyn et al., 2013). Benefits of hypothermia (which remain poorly known) should be sufficiently high to compensate for these costs.

## Conclusions

In short, our results suggest that lizards adjust their thermal response to an immune challenge according to the costs/benefits balance, leading to elevational variation in the thermal response to the activation of the immune system. Non-alpine lizards showed decreased ${T}_{\mathrm{pref}}$ in response to immune challenge, presumably in order to save energy. However, alpine lizards prioritized heat gain, and maintained their ${T}_{\mathrm{pref}}$. This matches with increased costs of non-thermoregulation in alpine lizards, due to the reduced window for reproduction in the environment where they have evolved.

## Supplemental Information

10.7717/peerj.1972/supp-1 Data S1 Raw data

## Additional Information and Declarations

### Field Study Permissions

The following information was supplied relating to field study approvals (i.e., approving body and any reference numbers): The experiments comply with the current laws of Spain, and were performed in accordance with the Junta de Andalucía research permits issued to the authors FJZC, SR and GMR (references GMN/GyB/JMIF and ENSN/JSG/JEGT/MCF).

### Data Availability

The following information was supplied regarding data availability: Figshare: http://figshare.com/articles/Elevational_variation_in_body_temperature_response_to_immune_challenge_in_a_lizard/1597645.
